# An Analysis of the Effect of Abrasive/Tool Wear on the Ductile Machining of Fused Silica from the Perspective of Stress

**DOI:** 10.3390/mi13060820

**Published:** 2022-05-25

**Authors:** Ming Li, Xiaoguang Guo, Song Yuan, Bingyao Zhao, Yongnian Qi, Shuohua Zhang, Dongming Guo, Ping Zhou

**Affiliations:** Key Laboratory for Precision and Non-Traditional Machining Technology of Ministry of Education, School of Mechanical Engineering, Dalian University of Technology, Dalian 116024, China; lmzdg@mail.dlut.edu.cn (M.L.); guoxg@dlut.edu.cn (X.G.); yuansong@mail.dlut.edu.cn (S.Y.); zby666666@mail.dlut.edu.cn (B.Z.); yunai2384@163.com (Y.Q.); dutzfl@163.com (S.Z.); guodm@dlut.edu.cn (D.G.)

**Keywords:** fused silica, sph, wear, edge radius, ductile machining, Flamant’s formula

## Abstract

Understanding the influence mechanism of abrasive/tool wear on machining is the key to realize high-efficiency ultra-precision machining of fused silica. To explore the effect of abrasive/tool wear on ductile machining, the smoothed particle hydrodynamics (SPH) cutting models with different edge radii are established. Through the analysis of equivalent rake angle, hydrostatic pressure, cutting force and maximum principal stress with the Flamant’s formula, the influence of edge radii on ductile-brittle transition (DBT) is discussed for the first time. The simulation results show that when the edge radius increases from less to larger than the cutting depth, the equivalent rake angle changes from positive to negative, and the maximum hydrostatic pressure gradually increases, which is beneficial to promote the ductile processing. Meanwhile, with the rise of edge radius (i.e., abrasive/tool wear), both the cutting force and crack initiation angle increase, while the friction coefficient and normalized maximum principal decrease. When the value of normalized maximum principal stress exceeds 2.702, the crack in the workpiece begins to initiate, and its initiation angle calculated by the Flamant’s formula is in good agreement with the simulation results as well as less than 50°. Finally, the nano-scratch experiment was carried out, and the material removal mechanism and friction coefficient *f* similar to the simulation were obtained, which further proved the accuracy of SPH model. This study is meaningful for understanding the effect of abrasive/tool wear on the removal mechanism of brittle materials and improving the quality and efficiency of cutting and grinding.

## 1. Introduction

Fused silica is widely available for aerospace (observation window of spacecraft), semiconductor (wafer carrier), and other fields due to its high strength, high temperature resistance, electrical insulation, etc. Nevertheless, as a typical brittle material, it is easy to form surface and subsurface cracks during grinding or cutting [1,2,3]. How to analyze the fracture characteristics of hard and brittle materials and achieve high-quality and high-efficiency machining is an urgent problem to be solved.

At present, ductile-mode machining has become an ultra-precision processing method for brittle materials to avoid fracture defects [4,5]. Studies show that when the cutting depth is very small, ductile chips will be formed in brittle materials during processing, and the material removal mode is ductile machining [6,7]. Abrasive or tool wear is almost inevitable in ultra-precision machining of hard and brittle materials [8,9]. Song et al. [10] carried out laser assisted machining (LAM) experiments of fused silica with polycrystalline diamond tool (PCD), polycrystalline cubic boron nitride tool (PCBN), and tinned Al_2_O_3_/TiC ceramic tool, respectively, clarified the wear mechanism of the three tools, and pointed out that severe wear will affect the life of the tool. Chen et al. [11] found that the passivation of the grinding wheel will increase the friction between the grinding wheel and the workpiece, and the cutting force state and passivation degree of the grinding wheel can be reflected through the noise spectrum during processing. Zhou et al. [12] believed that diamond abrasive wear is the main form of grinding wheel wear, and they found that with the intensification of grinding wheel wear, the surface roughness and subsurface damage depth first decreased, then fluctuated and increased slowly. However, the influence mechanism of abrasive/tool wear on ductile machining of fused silica is not clear.

In the ductile machining of brittle materials, the uncut chip thickness is close to the abrasive/tool edge radius, which is at submicron level [13,14]. The wear degree of abrasive or tool can usually be regarded as the passivation degree of the tip, that is, the size of tip radius. The edge radius will restrict the flow of chips, and its influence on the ductile processing cannot be ignored [15,16]. Yan et al. [17] analyzed the sub-micron orthogonal cutting process of single crystal silicon by finite element method (FEM), and they pointed out that an important factor affecting the ductility of silicon is hydrostatic pressure, which is dominant in the material in front of the tool. Cai et al. [18] used the molecular dynamics (MD) method to simulate the influence of tool edge radius on the cutting process of single crystal silicon, and found that a larger tool edge radius is conducive to the ductile domain processing. However, the MD model has a small scale and a large amount of calculation, which makes it difficult to simulate cracks. To analyze the influence of edge radius (i.e., abrasive/tool wear) on ductile processing of hard and brittle materials more intuitively, a sub-micron simulation method that can describe fracture characteristics as needed. As a typical meshless method, the smoothed particle hydrodynamics (SPH) method has been widely used in the study of cracks in hard and brittle materials [19,20,21]. Unfortunately, there is no detailed report on the influence of edge radius on ductile processing using the SPH method.

In this paper, a sub-micron cutting SPH model of fused silica considering edge radius was successfully built. In addition, a nano-scratch experiment was performed to analyze the material removal mechanism and to verify the accuracy of the SPH model. By discussing the equivalent rake angle, hydrostatic pressure and cutting force during processing, and combining with the analysis of the maximum principal stress, the influence of edge radius (i.e., abrasive/tool wear) on the ductile processing of fused silica is obtained for the first time. The research is helpful for the optimization of abrasive/tool and the understanding of the removal mechanism of brittle materials.

## 2. SPH Methodology of Fused Silica

### 2.1. The Basic Theory of the SPH Method

As a mesh-free technology, the SPH method can avoid the problem of mesh distortion, and it is good at dealing with large deformation problems [22]. The basis of SPH is interpolation theory [23]. The interpolation function is able to describe the interaction of each particle, and the core estimation of any point in the quantity field is given. The conservation law of continuum dynamics is transformed from the form of differential equation to the form of integral, and then to the form of summation.

The physical quantity f(*r*) is approximated by the kernel function as:

(1) $$f\left(r\right)=\int_{\Omega }W\left(r-x,\;h\right)dx,$$

in the formula, *W* and *h* are the kernel function and smooth length, respectively. *r* is the position vector of the fixed point, and *x* is the position vector of the variable point.

For a smooth function *W* using the cubic B-spline function, it can be expressed as:

(2) $$W\left(r-x,\;h\right)=W\left(R,\;h\right){=a}_{d}\{\begin{array}{c} \frac{2}{3}-{\;R}^{2}+\frac{R^{3}}{2},\;0\;\leq \;R\;<\;1\\ \frac{{\left(2-R\right)}^{3}}{6},\;1\;\leq \;R\;<\;2\\ 0,\;R\;\geq \;2 \end{array},$$

where, *d* is the spatial dimension, and in one-dimensional, two-dimensional, and three-dimensional space, *a*_d_ is 1/*h*, 15/(7π*h*^2^) and 3/(2π*h*^3^), respectively.

**Figure 1:**
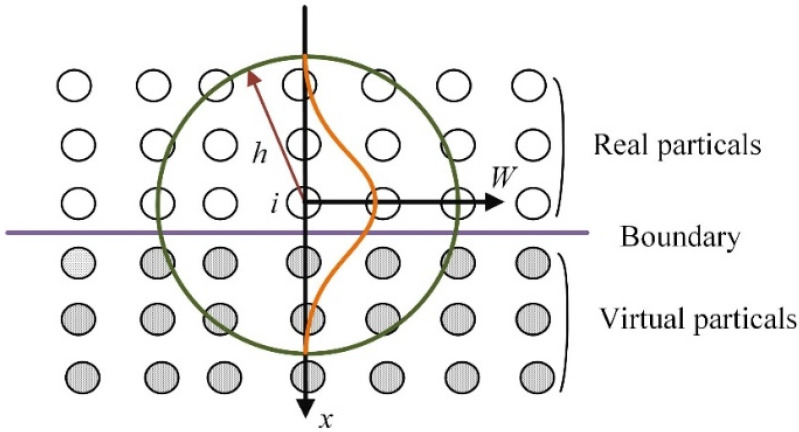
The schematic of boundary virtual particles: the green circle is the search area of evaluation point *i*, and the orange line is the distribution of the smooth function *W*.

It should be noted that in the field of solid processing, the asymmetry of particle distribution at the boundary will cause the kernel function to be truncated, which will lead to solution errors. In order to avoid this problem, the virtual particle method is usually used to constrain the solid wall boundary [24]. The essence of the virtual particle method is to discretize the boundary of the solid wall into multiple rows of virtual particles. As shown in Figure 1, within the range of the smooth length *h* of the evaluation point *i*, when searching for the real particles of the nearest neighbors, the virtual particles should also be searched [25]. In this paper, the virtual particle model is used to calculate the cutting process of fused silica.

### 2.2. SPH Modeling

**Figure 2:**
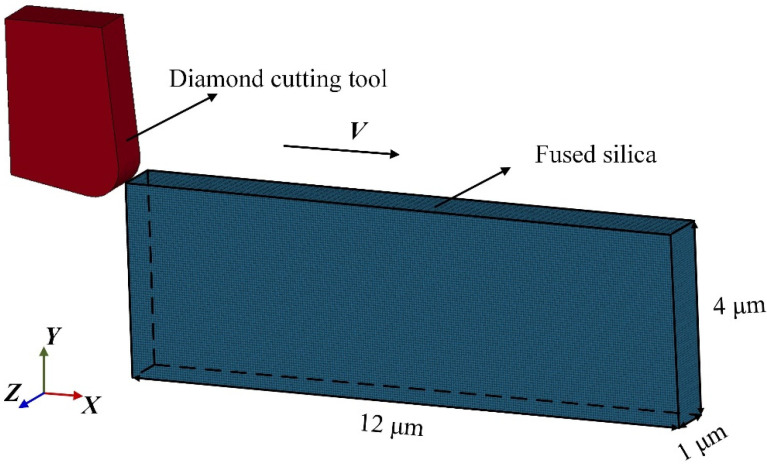
Cutting model of fused silica.

Figure 2 shows the sub-micron cutting SPH model of fused silica. All simulation programs are solved in the software ls-dyna_mpp_s_R12. In this model, the elastic modulus of the diamond tool far exceeds that of fused silica. Therefore, the tool is modelled as a rigid body with an elastic modulus of 1140 GPa, a density of 3.52 g·cm^−3^, and a Poisson’s ratio of 0.07. The material properties of the tool are defined by the keyword MAT_RIGID, and the movement of the tool in the Y and Z directions and the rotation around the X, Y, and Z axes are constrained. The rake angle of the diamond tool is 5°, the relief angle is 8°, and the edge radius is 0.5 μm. To generate the mesh of the diamond tool, the volume tetra command can be applied in the software Hypermeshv14.0, and the total number of tetrahedral meshes is 5196. The size of the workpiece is 12 μm × 4 μm × 1 μm, containing 144,000 SPH particles, which are obtained through the command “SPH generation”. The virtual particle method is used to control the boundary of the workpiece. The movement in the Y direction of the workpiece bottom, the Z direction of the workpiece sides, and the X direction of the workpiece back are restricted by the keyword “Boundary_SPH_symmetry_plane”. In addition, a velocity load in the X direction can be applied to the diamond tool through the keyword “Boundary_prescribed_motion_rigid”. The cutting speed is 40 m/s, the cutting depth is 0.3 μm, and the maximum cutting length is 10 μm. In the simulation, the constitutive model is used to describe the stress–strain relationship of materials, and the accuracy of material constitutive model parameters directly affects the simulation results. Johnson–Holmquist ceramics (JH-2) model is widely used in the cutting or grinding simulation of hard and brittle materials such as glass and ceramics. This model is suitable for simulating large deformations of materials. In order to study the kinetic behavior of DBT during the processing of fused silica, a JH-2 material model is adopted in this paper. The specific parameters are given in Table 1 [26]. The damage model in the JH-2 constitutive model is the same as that in the Johnson–Cook, and the damage variable *D* can be expressed as:

(3) $$D=\sum \left(\Delta \varepsilon_{p}/\varepsilon_{p}^{f}\right),$$

in the formula, $\Delta \varepsilon_{p}$ is the integral of the effective plastic strain in a single cycle, $\varepsilon_{p}^{f}$ is the crushing plastic strain of the material under a certain pressure, and its expression is:

(4) $$\varepsilon_{p}^{f}=D_{1}(p^{*}{+\sigma }_{t,m}^{*})/D_{2},$$

where, *D*_1_ and *D*_2_ are the material damage parameters, p* is the dimension hydrostatic pressure, and $\sigma_{t,m}^{*}$ is the dimension maximum hydrostatic tensile stress.

When the material is not damaged, the state equation of the material in the JH-2 constitutive model is:

(5) $${p=K}_{1}{\mu +K}_{2}\mu^{2}{+K}_{3}\mu^{3},$$

where, *K*_1_ (GPa) is the bulk modulus, *K*_2_ (GPa) and *K*_3_ (GPa) are the material constants, *p* (GPa) is the hydrostatic pressure, and *μ* is the volume strain.

The strength model of JH-2 correlates with the damage (*D*) of the material. The dimensional equivalent stress can be expressed as:

(6) $$\{\begin{array}{c} \sigma_{i}^{*}{=A(p}^{*}{+\sigma }_{t,m}^{*}{)}^{N}[1+\mathrm{Cln}(\overset{\cdot }{\varepsilon }/{\overset{\cdot }{\varepsilon }}_{0})],\;D=0\\ \sigma_{f}^{*}{=B(p}^{*}{)}^{M}[1+\mathrm{Cln}(\overset{\cdot }{\varepsilon }/{\overset{\cdot }{\varepsilon }}_{0})],\;D=1 \end{array},$$

where, *A*, *B*, *C*, *M*, and *N* are material constants, $\overset{\cdot }{\varepsilon }$ is the real strain rate, and $\varepsilon_{0}$ is the reference strain rate.$\;p^{*}=p/p_{HEL}$. $\sigma_{t,m}^{*}=\sigma_{t,m}/p_{HEL}$. $p_{HEL}$ (GPa) is the pressure component of the material Hugoniot elastic limit ($\sigma_{HEL}$), and $\sigma_{t,m}$ (GPa) is the maximum hydrostatic tensile stress of the material.

## 3. Nano-Scratch Experiment of Fused Silica

**Figure 3:**
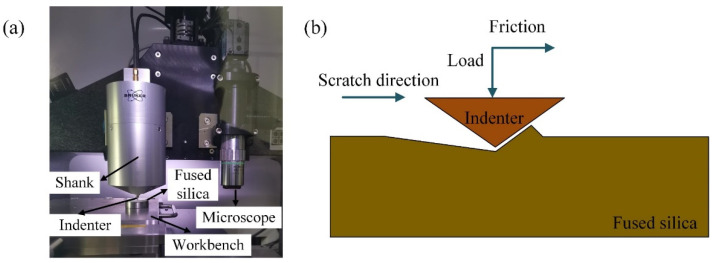
The device (**a**) and principle (**b**) of nano-scratch experiment of fused silica.

To analyze the material removal mechanism of fused silica and to verify the accuracy of the SPH model, a variable load nano-scratch experiment was carried out. The surface roughness *R_a_* (20 nm) of fused silica is measured by KEYENCE vk-x250 laser confocal microscope, which meets the requirements of the experiment. Additionally, the sample is columnar fused silica with the size of *φ* 25 mm × 2 mm. A HYSITRON Ti 950 triboindenter nano-indenter and a diamond Berkovich indenter were adopted in the experiment. The tip radius of the Berkovich indenter was approximately 0.5 μm. The scratch length was 300 μm, the travelling speed was 8 μm /s, and the maximum load was 200 mN. The scratch experimental device and the basic principle are given in Figure 3.

## 4. Results and Discussion

### 4.1. Ductile Domain Processing

Bifano [6] studied the ductile process from the energy change mechanism of hard–brittle materials. According to the energy calculation formula, when the cutting depth *d* exceeds a critical value *d_c_*, the brittle material tends to brittle fracture during machining, while *d* < *d_c_*, the plastic deformation prevails.

**Figure 4:**
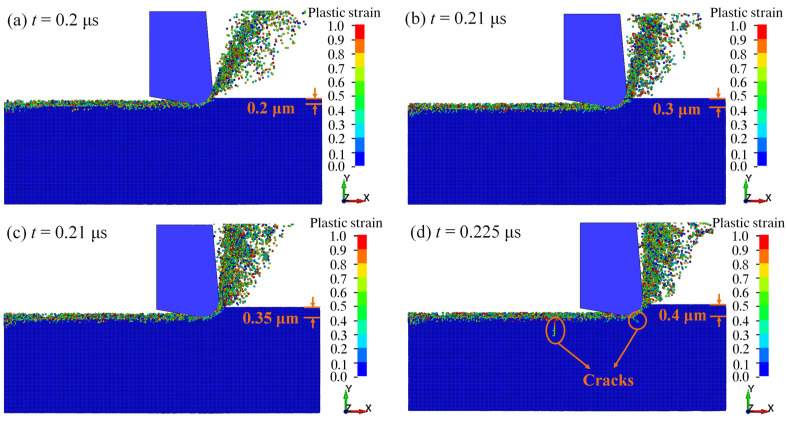
The distribution of plastic strain at the cutting depth of (**a**) 0.2 μm, (**b**) 0.3 μm, (**c**) 0.35 μm, and (**d**) 0.4 μm.

Figure 4 shows the plastic strain of fused silica under different processing depth *a_p_* at the tool edge radius *r* of 0.6 μm. When the SPH particle is separated from the workpiece, its damage value (*D*) reaches 1, and then it can be considered as invalid [27]. In order to better observe the distribution of SPH particles in the workpiece, some invalid particles that are separated from the workpiece have been deleted. When *a_p_* = 0.2 μm (Figure 4a), 0.3 μm (Figure 4b), and 0.35 μm (Figure 4c), there is no obvious subsurface crack generated during the cutting, indicating that the material has undergone ductile domain processing on a large extent. When *a_p_* = 0.4 μm (Figure 4d), obvious median cracks appear on the subsurface. Thus, the critical depth of the DBT of fused simulated silica is considered as 0.4 μm.

**Figure 5:**
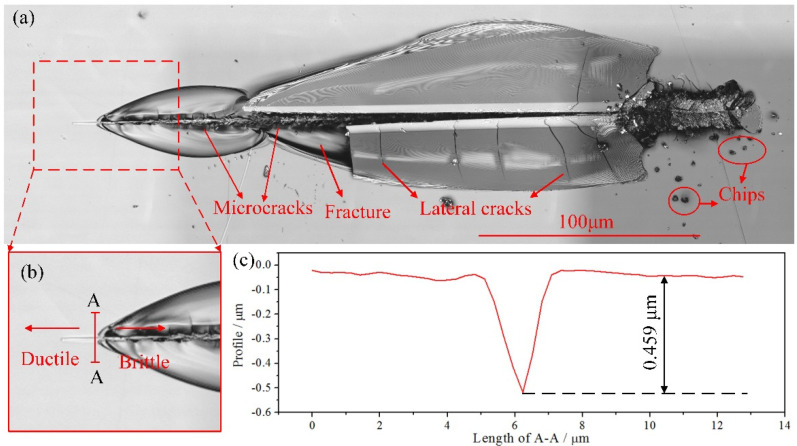
The complete (**a**) and partial (**b**) scratch morphology, and profile at A-A (**c**).

Figure 5 presents the scratch morphology of fused silica observed with a laser confocal microscope. Due to the variable cutting depth scratch process (Figure 5a), the scratch depth is very small at the initial moment, and the workpiece can be machined in the ductile region. Then, as the scratch depth exceeds the critical depth of the DBT, microcracks appear on the workpiece. With the increase of scratch depth, lateral cracks, fractures and chips occur, and the removal process changes to brittle removal. It can be seen from Figure 5b that when the scratch does not reach A-A, there is no obvious crack on the surface of the workpiece, which is manifested as ductile domain processing. When the scratch exceeds A-A, the workpiece is removed in the form of a brittle fracture. Therefore, it can be considered that a plastic-brittle transition has occurred at A-A. The profile at A-A is shown in Figure 5c. And the critical depth of the DBT of fused silica is approximately 0.459 μm. Obviously, it is close to the simulated result (0.4 μm). This proves the correctness of the SPH model to a certain extent.

### 4.2. Equivalent Rake Angle

Previous studies have shown that the processing scale of the ductile domain of brittle materials is usually 1 μm or less, which belongs to the submicron level [13,14]. The diamond tool edge radius is approximately 20 nm~1 μm. Thus, the influence of the edge radius on the ductility domain processing cannot be neglected. An important effect of the tool edge radius is reflected in the equivalent rake angle *γ_e_*, which is considered to be the rake angle for a given uncut chip thickness and tool edge radius [28]. Usually, it can be expressed as:

(7) $$\gamma_{e}=\{\begin{array}{c} \gamma ,\;r\leq d\\ -\sin^{-1}\left(1-\frac{d}{r}\right),\;r>d \end{array},$$

where, *γ* is the rake angle of the tool, *r* is the tool edge radius and *d* is the cutting depth.

**Figure 6:**
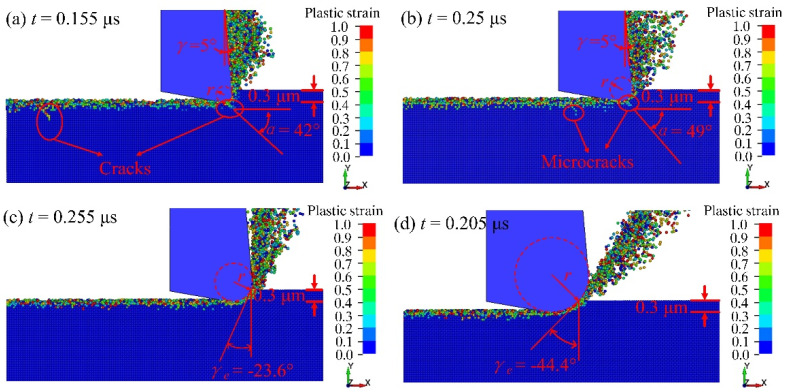
The distribution of plastic strain at the tool edge radius of (**a**) 0.2 μm, (**b**) 0.3 μm, (**c**) 0.5 μm, and (**d**) 1.0 μm.

Figure 6 presents the plastic strain of fused silica under different edge radius *r* when the processing depth *a_p_* is 0.3 μm. *α* is the angle between the crack initiation direction and the surface of the workpiece. It can be seen from Figure 6a that the equivalent cutting rake angle *γ_e_* is 5° when the tool edge radius is less than the depth of cut. At this time, obvious cracks occur in the workpiece (*α* = 45°), indicating that the material is brittlely removed. As shown in Figure 6b, the *γ_e_* is also 5° when the tool edge radius is equal to the cutting depth, while only a few micro-cracks appear on the subsurface (α = 55°). The *γ_e_* becomes −23.6° when the tool edge radius is 0.5 μm, exceeding the cutting depth, and there are no obvious cracks on the surface and subsurface of the material (Figure 6c). The value of the negative equivalent rake angle further increases, and it becomes −44.4° when the radius of the tool edge is 1.0 μm and the material has undergone ductile processing (Figure 6d).

The simulation results show that as the edge radius increases, the equivalent rake angle changes from a positive value to a negative value, and it continues to rise, making the ductile domain processing of the material easier to achieve. Zhou et al. [12] found that when the wear of a diamond grinding wheel is small, with the increase of wear, the subsurface damage depth of fused silica will decrease. This is consistent with the conclusion of simulation.

### 4.3. Hydrostatic Pressure

**Figure 7:**
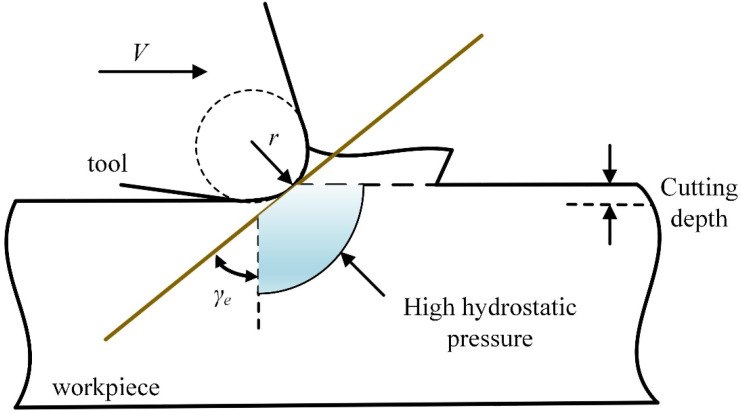
Influence of tool edge radius on ductile cutting.

Usually, the tool edge radius easily makes the equivalent rake angle behave as a negative value, which causes the material in front of the tool to be squeezed and to form compressive stress similar to hydrostatic pressure, as shown in Figure 7 [29]. In fact, hard and brittle materials will show obvious plastic flow behavior under higher hydrostatic pressure [30].

**Figure 8:**
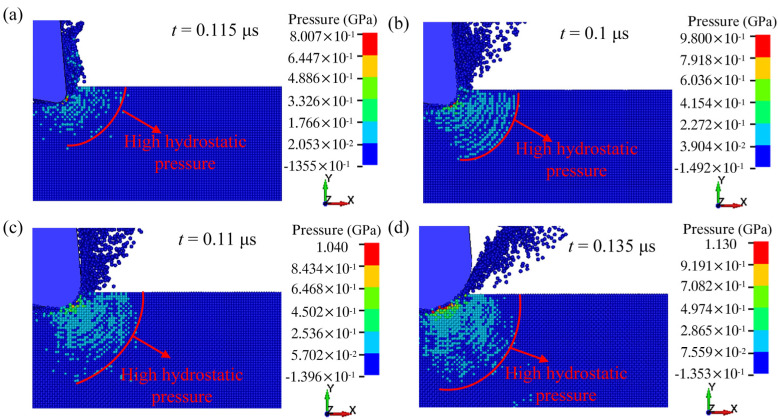
The distribution of hydrostatic pressure at the tool edge radius of (**a**) 0.2 μm, (**b**) 0.3 μm, (**c**) 0.5 μm, and (**d**) 1.0 μm.

Figure 8 shows the hydrostatic pressure distribution under different tool edge radii. When *r* = 0.2 μm (Figure 8a), significant high hydrostatic pressure is generated at the front of the tool tip. When *r* = 0.3 μm (Figure 8b), the area of high hydrostatic pressure slightly increases. When *r* increases from 0.5 μm to 1.0 μm (Figure 8c,d), the high hydrostatic pressure expands forward and downward.

**Figure 9:**
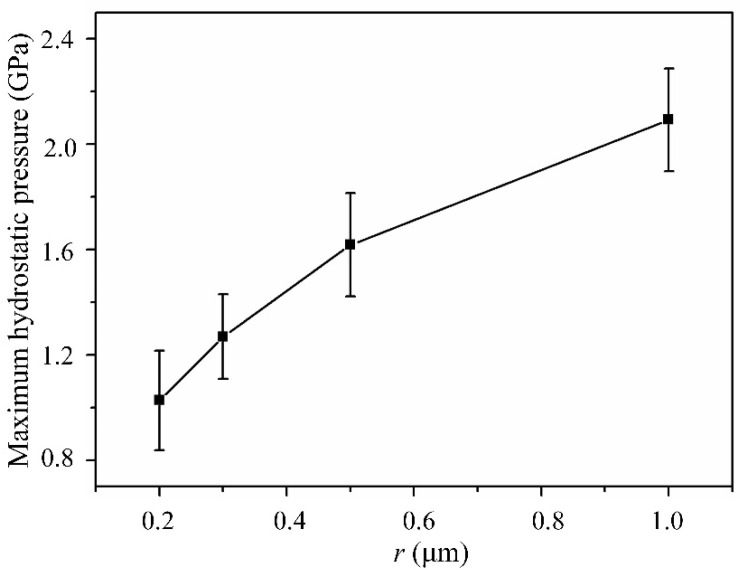
The maximum hydrostatic pressure under different tool edge radii.

Figure 9 shows the maximum hydrostatic pressure values under different tool edge radii. It is easy to observe that the maximum hydrostatic pressure increases as the tool edge radius increases. This is consistent with the previous analysis, indicating a larger tool edge radius is beneficial to ductile cutting.

### 4.4. Cutting Force Analysis

**Figure 10:**
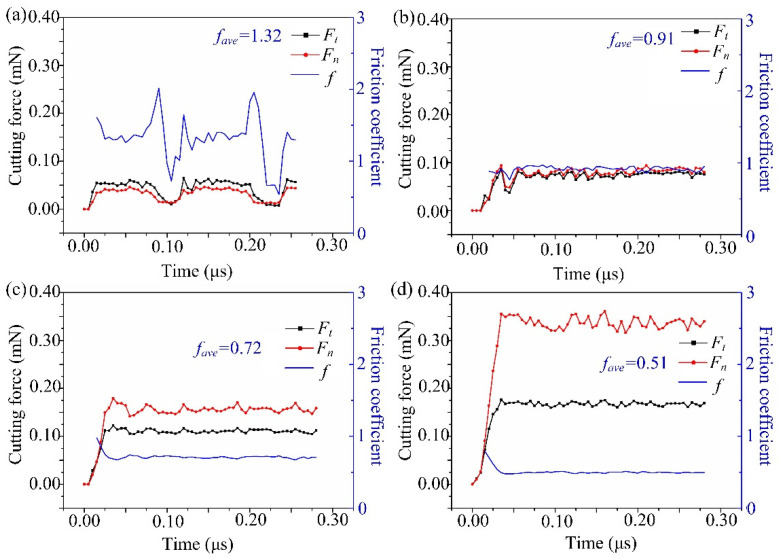
Simulated cutting force and friction coefficient at the tool edge radius of (**a**) 0.2 μm, (**b**) 0.3 μm, (**c**) 0.5 μm, and (**d**) 1.0 μm.

Figure 10 shows the cutting force and friction coefficient under different edge radii. The friction coefficient *f* is the ratio of *F_t_* to *F_n_*, and its average value is *f_ave_*. When *r* = 0.2 μm (Figure 10a), the tangential cutting force *F_t_* is greater (*f_ave_* > 1), and the cutting force is obviously unstable during the cutting process. This is because the removal form at this time is a brittle fracture, as shown in Figure 6a. When *r* = 0.3 μm (Figure 10b), the cutting force fluctuates greatly in the initial stage, and then it is flat. Meanwhile, the normal cutting force *F_n_* is basically the same as *F_t_* (*f_ave_* ≈ 1). When *r* = 0.5 μm (Figure 10c) and 1.0 μm (Figure 10d), *F_n_* obviously exceeds *F_t_* (*f_ave_* < 1), and the fluctuations are small. Thus, the material is mainly removed from the ductile domain.

The simulation shows that with the increase of *r*, the contact area between the material and the tool rises, which causes the rise of the force to resist the friction and the deformation of the workpiece. At the same time, as *r* rises, the tool downward squeezing effect increases, and the friction coefficient decreases.

### 4.5. Maximum Principal Stress Analysis

**Figure 11:**
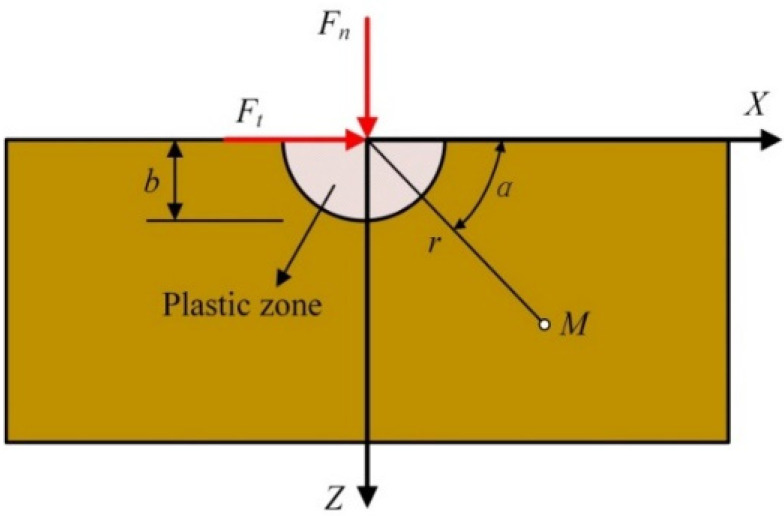
Cutting force acting on a half-plane.

For the loading of an isotropic elastic substrate, the Flamant’s problem is that the force perpendicular to the edge of the half-plane (plane stress state) spreads radially in the substrate [31,32]. According to the Flamant’s problem, both the normal force *F_n_* and the tangential force *F_t_* will cause a stress field in the elastic area in the cutting process of hard and brittle materials, as shown in Figure 11. The radius of the plastic zone is *b*.

The expression of the stress field *α_ij_* caused by *F_n_* is:

(8) $$\{\begin{array}{c} \alpha_{\mathrm{rr}}{=2F}_{n}\sin\alpha /(\pi r)\\ \alpha_{\alpha \alpha }=0\\ \alpha_{r\alpha }=0 \end{array}.$$

The expression of stress field *β_ij_* caused by *F_t_* is:

(9) $$\{\begin{array}{c} \beta_{\mathrm{rr}}{=2F}_{t}\cot\alpha /(\pi r)\\ \beta_{\alpha \alpha }=0\\ \beta_{r\alpha }=0 \end{array}.$$

Therefore, the stress field *σ_ij_* caused by the cutting force in the polar coordinate system (*r*, *α*) can be expressed as:

(10) $$\{\begin{array}{c} \sigma_{\mathrm{rr}}{=\alpha \;}_{\mathrm{rr}}{+\beta }_{\mathrm{rr}}{=2F}_{n}{\sin\alpha /(\pi r)+2F}_{t}\cos\alpha /(\pi r)\\ \sigma_{\alpha \alpha }{=\alpha }_{\alpha \alpha }{+\beta }_{\alpha \alpha }=0\\ \sigma_{r\alpha }{=\alpha }_{r\alpha }{+\beta }_{r\alpha }=0 \end{array}.$$

Converting the stress components in the polar coordinate system (*r*, *α*) to the rectangular coordinate system XZ, *σ_xx_*, *σ_zz_* and *σ_zx_* can be obtained. Then, the maximum principal stress σ_1_ can be calculated by the following formula:

(11) $$\sigma_{1}{=(\sigma }_{\mathrm{xx}}{+\sigma }_{\mathrm{zz}})/2+\sqrt{{{(\sigma }_{\mathrm{xx}}-{\;\sigma }_{\mathrm{zz}})}^{2}{/4+\sigma }_{\mathrm{zx}}^{2}}.$$

**Figure 12:**
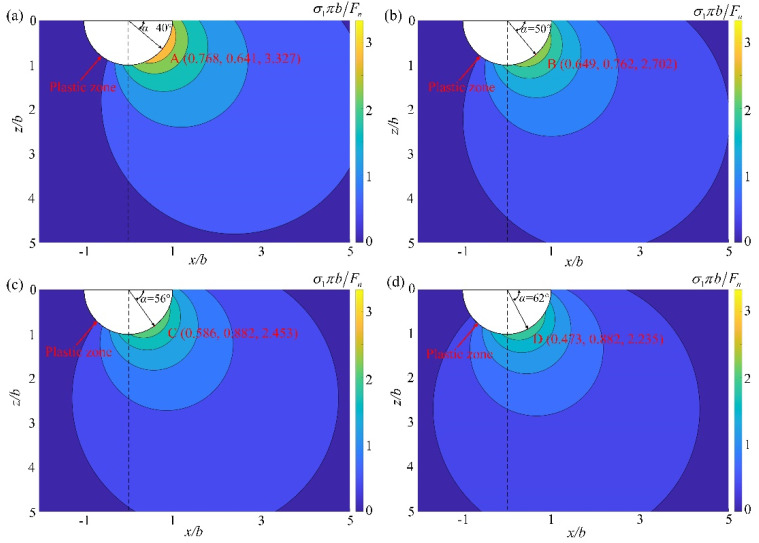
Normalized maximum principal stress at the tool edge radius of (**a**) 0.2 μm, (**b**) 0.3 μm, (**c**) 0.5 μm, and (**d**) 1.0 μm.

According to fracture mechanics, when the maximum principal stress exceeds the fracture strength, cracks begin to initiate. Thus, it is necessary to analyze the maximum principal stress *σ*_1_ of hard and brittle materials in the XZ plane. In order to facilitate the analysis, the maximum principal stress *σ*_1_ is normalized. Additionally, the normalized maximum principal stress *σ*_1_*πb*/*F_n_* is closely related to the friction coefficient *f*. When the cutting depth is 0.3 μm, the distribution of the maximum principal stress under different tool edge radii in the rectangular coordinate system is shown in Figure 12. Points A, B, C, and D are the peak values of the normalized maximum principal stress in Figure 12a–d, respectively. Obviously, as the tool edge radius increases, the peak value of normalized maximum principal stress decreases. According to the simulation results, cracks will occur when *r* ≤ 0.3 μm. Then, the normalized maximum principal stress that can produce cracks should exceed 2.702 (Figure 12b). In addition, at the peak of the maximum principal stress, cracks are the most prone to initiation. As the tool edge radius increases from 0.2 μm to 0.3 μm, the crack initiation angle *α* increases from 40° to 50°. This is consistent with the simulation results (Figure 6a,b), which further illustrates the correctness of the simulation.

### 4.6. Verification of the SPH Simulation

**Figure 13:**
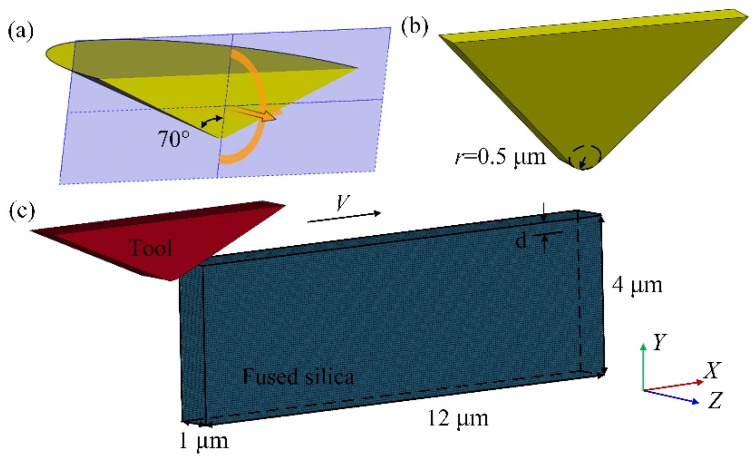
The conical indenter section (**a**), triangular prism diamond tool (**b**) and SPH cutting model (**c**).

The indenter used in the nano-scratch experiment is a Berkovich indenter, which can usually be simplified to a cone with a half cone angle of 70°. A cross-sectional view of the cone along the midline is shown in Figure 13a. To make the tool used in the simulation and the indenter used in the experiment as similar as possible, a triangular prism diamond tool with a thickness of 1 μm and a tip radius of 0.5 μm was established (Figure 13b). The established cutting model is shown in Figure 13c, and the modelling method is the same as above (*2.2*). The cutting speed is 40 m/s, the cutting length is 6 μm, and the cutting depth *d* is 0.3 μm, 0.4 μm, and 0.5 μm, respectively.

**Figure 14:**
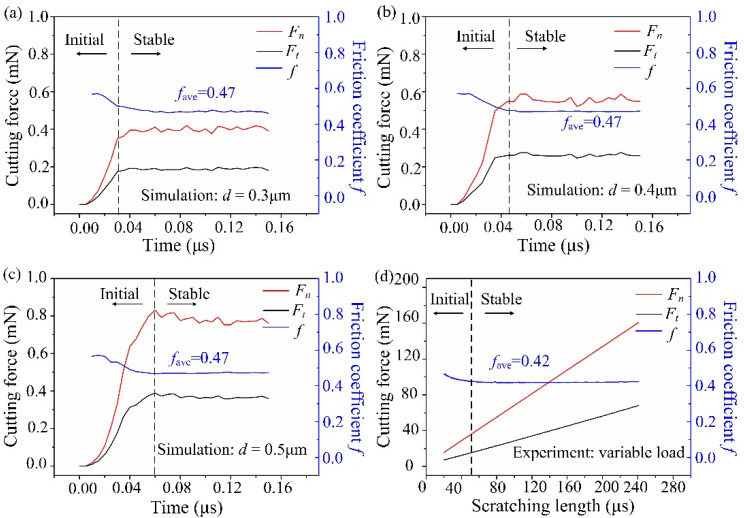
The simulated (**a**–**c**) and experimental (**d**) normal cutting force (*F_n_*), tangential cutting force (*F_t_*), and friction coefficient *f* during the scratching process of fused silica.

The normal cutting force *F_n_*, tangential cutting force *F_t_*, and friction coefficient *f* during the simulation and the experiment are shown in Figure 14. The simulated cutting process can be divided into the initial and the stable cutting stage (Figure 14a–c). In the initial cutting stage, *F_n_* and *F_t_* increase rapidly, and *f* is not stable. In the stable cutting stage, *F_n_* and *F_t_* fluctuate little, and *f* is stable around 0.47. A similar phenomenon was also observed in the nano-scratch experiment of fused silica (Figure 14d). In the stable stage of the nano-scratch experiment, the average value of *f* is approximately 0.42, which is very close to the simulated results. This proves that the simulation results are reliable. Also, it should be noted that the cutting speed of the simulation model (40 m/s) far exceeds the scratch experiment (8 μm/s). Additionally, limited to the scale of simulation, it is difficult to simulate the actual machining process, which may be the reason for the certain difference between the simulation and the experimental results. With the rapid progress of computer technology, large-scale simulation close to the actual working condition may be an important direction of future research work.

## 5. Conclusions

In this paper, a submicron cutting SPH model of fused silica is built. The influence of the edge radius (i.e., abrasive/tool wear) on ductile processing is studied from the perspectives of effective rake angle, hydrostatic pressure, cutting force, and maximum principal stress. The following conclusions are obtained:

(1) The material removal characteristic and friction coefficient *f* simulated by the built SPH model had a good agreement with the nano-scratch experiment, and the crack initiation angle obtained from the simulation results was close to that calculated by Flamant’s formula, which verified the accuracy of the SPH model.
(2) By comparing the results of the SPH model and Flamant’s formula, it was found that the stress field based on approximate concentrated force can be used for the critical depth analysis of DBT. In addition, the influence of edge radius can be reflected by the change of the friction coefficient. Furthermore, the smaller the friction coefficient, the smaller the possibility of crack.
(3) As the edge radius increases, the negative value of the equivalent rake angle, maximum hydrostatic pressure, cutting force, and crack initiation angle increase, while the friction coefficient and the normalized maximum principal stress decrease. The study in this paper is helpful to understand the influence mechanism of abrasive/tool wear on ductile machining, and it provides a theoretical basis for the optimization of ultra-precision cutting or grinding processes of hard and brittle materials.

## Figures and Tables

**Table 1:** JH-2 model parameters of fused silica.

Parameters	Value	Parameters	Value
Density	2.20 g·cm^−3^	Complete strength (*N*)	0.75
Shear modulus	31 GPa	Hug elastic limit (HEL)	9 GPa
Tensile strength	0.05 GPa	Elastic strain (*D*_1_)	0.053
Standard strength (*A*)	0.93	Elastic strain (*D*_2_)	0.85
Fracture strength (*B*)	0.088	First pressure coefficient (*K*_1_)	45.4 GPa
Strain rate strength (*C*)	0.003	Second pressure coefficient (*K*_2_)	−138 GPa
Pressure index (*M*)	0.29	Elastic constant (*K*_3_)	290 GPa

## Data Availability

Not applicable.
